# Long-pulsed nd: YAG laser treatment of nail psoriasis: clinical and ultrasonographic assessment

**DOI:** 10.1007/s00403-024-03036-7

**Published:** 2024-06-08

**Authors:** Mohamed Ahmed Salem El-Basiony, Mohamed Hussein Medhat El-Komy, Nevien Ahmed Samy, Dalia Gamal Aly, Hala El-Gendy, Mohamed Fouad Abdel Salam Hassan, Hagar El Sayed, Mohamed Mohsen Soliman

**Affiliations:** 1 Department of Dermatology and Venereology, Medical Research and Clinical Studies Institute, National Research Centre, Giza, Egypt; 2 Department of Dermatology, Kasr Al-Ainy Psoriasis Unit, Faculty of Medicine, Cairo University, Giza, Egypt; 3 Dermatology Unit, Department of Medical Applications of Lasers, National Institute of Laser Enhanced Sciences, Cairo University, Giza, Egypt; 4 Department of Internal Medicine and Rheumatology, Faculty of Medicine, Cairo University, Giza, Egypt

**Keywords:** Nd:YAG laser, Ultrasonography, Target NAPSI, Nail psoriasis

## Abstract

Nail psoriasis is a chronic, inflammatory condition which is difficult to treat, linked with greater psoriasis severity, and may be associated with anxiety and significant functional impairment of the quality of life. The 1064 nm Nd: YAG laser was reported to yield satisfactory results in the treatment of nail psoriasis.

The aim of the study was to assess the clinical and ultrasonographic efficacy of long-pulsed 1064 nm Nd: YAG laser in the treatment of fingernail psoriasis and compare its effect to control fingernails.

This intra-patient randomized controlled trial analyzed 86 fingernails collected from 13 patients suffering from cutaneous and nail psoriasis. The nails were randomized into two groups. Group A was treated with Nd: YAG laser once monthly for three sessions while group B served as control. Assessment took place at baseline, 1 and 3 months after the last treatment session. For scoring, the 32-points target NAPSI scoring systems was used. Additionally, two blinded dermatologists’ score of improvement, patients’ pain assessment by visual analogue score and ultrasonographic assessment were all performed.

At the end of follow up, the medians of tNAPSI score, plate definition, matrix thickness, bed thickness and bed vascularity decreased significantly in the Nd: YAG laser treated group in comparison to baseline (*p = 0.001*, *0.006*, *0.039*, *< 0.001* and *0.010*, respectively). While, there was a non-significant reduction in median tNAPSI score in the control group at last follow up, however, ultrasonography recorded a significant reduction in the medians of plate definition, bed thickness and vascularity (*p = 0.002*, *0.011* and *0.033*, respectively) from the baseline. Comparison of the Nd: YAG laser and the control groups showed no significant difference from baseline regarding the medians of tNAPSI, tNAPSI percentile improvement, pits count, blinded evaluation of photographs and ultrasonographic assessments.

In conclusion, Nd: YAG laser showed clinical and ultrasonographic improvement in fingernail psoriasis. Ultrasonography is a useful noninvasive tool in diagnosing and monitoring the clinical and even the subclinical changes in nail psoriasis. Nail psoriasis although difficult to treat, may show spontaneous improvement.

## Introduction

Nail psoriasis (NP) is a chronic, difficult to treat clinical presentation of psoriasis, associated with greater severity of the disease, characterized by a higher risk and earlier onset of psoriatic arthritis. Accordingly, it can be painful, and may be associated with anxiety and depression with significant functional impairment and reduction of the quality of life [1].

The prevalence of NP varies widely and prevalence studies are rare. Augustin et al. (2010) reported 40.9% of 3531 German patients with psoriasis during the years 2005 and 2007 in a cross-sectional study complained of NP, Armesto et al. (2011) undertook a prospective case-control study on 661 Spanish patients with psoriasis between 2007 and 2009 and reported that 47.4% of them had NP and, El-Komy et al. (2020) conducted a retrospective single-center study on 2534 Egyptian patients with psoriasis during the period from 2015 to 2018 and found that nail involvement was present among 524 (20.7%) of the patients [2–4].

Treatment of NP is challenging as the clinical improvement takes time to be observed and treatment is often met with poor compliance [5]. Therapies include patient’s education, topical, intra-lesional and systemic treatments, including biological therapies. Therapies are selected according to the disease severity, presence of comorbidities, and the impact of psoriatic nail dystrophy on the patient’s quality of life due to impaired function, pain, and aesthetics [6].

Laser modalities gained much attention in the treatment of NP; due to their penetrability into nail bed and/or nail matrix [7]. The 595 nm pulsed dye laser results showed significant improvement in treatment of NP, but causes some pain and leaves transient petechiae, hyperpigmentation and purpura [8]. Also, the 1064 nm neodymium doped: Yttrium Aluminum Garnet (Nd: YAG) laser was used in the treatment of NP, based on selective photo-thermolysis of hemoglobin mainly, to target the dermal vasculature of the nail bed with satisfactory results [9–11].

High-frequency ultrasound (US) with power Doppler (PD) assessment provides details of nails’ soft tissue structures and microvasculature. Thickened nail bed and matrix, alteration of nail plate definition and nail bed vascularization are the major US manifestation of nail psoriasis [12].

The aim of this study was to assess clinically and by ultrasonography the efficacy of Nd: YAG laser in the treatment of fingernail psoriasis and to compare its effect with control fingernails.

## Patients and methods

### Subjects

This randomized controlled study included 13 psoriasis vulgaris patients with evidence of nail psoriasis affecting at least six fingernails, recruited from Kasr Al-Aini Psoriasis Unit (KAPU), Cairo University and the dermatology clinic of the National Research Center (NRC), Egypt. All patients received an explanation of the steps of the study and signed an informed consent to participate in the study. The study was approved by the ethical committee of the National Institute of Laser Enhanced Sciences (NILES), Giza, Egypt with reference number “NILES-EC-CU 23/12/27”.

Patients were excluded if they received any systemic therapies for psoriasis in the last 6 months or any topical treatments for the last two weeks. Other exclusion criteria included patients with any other chronic illnesses and patients with proven onychomycosis.

**Fig. 1:**
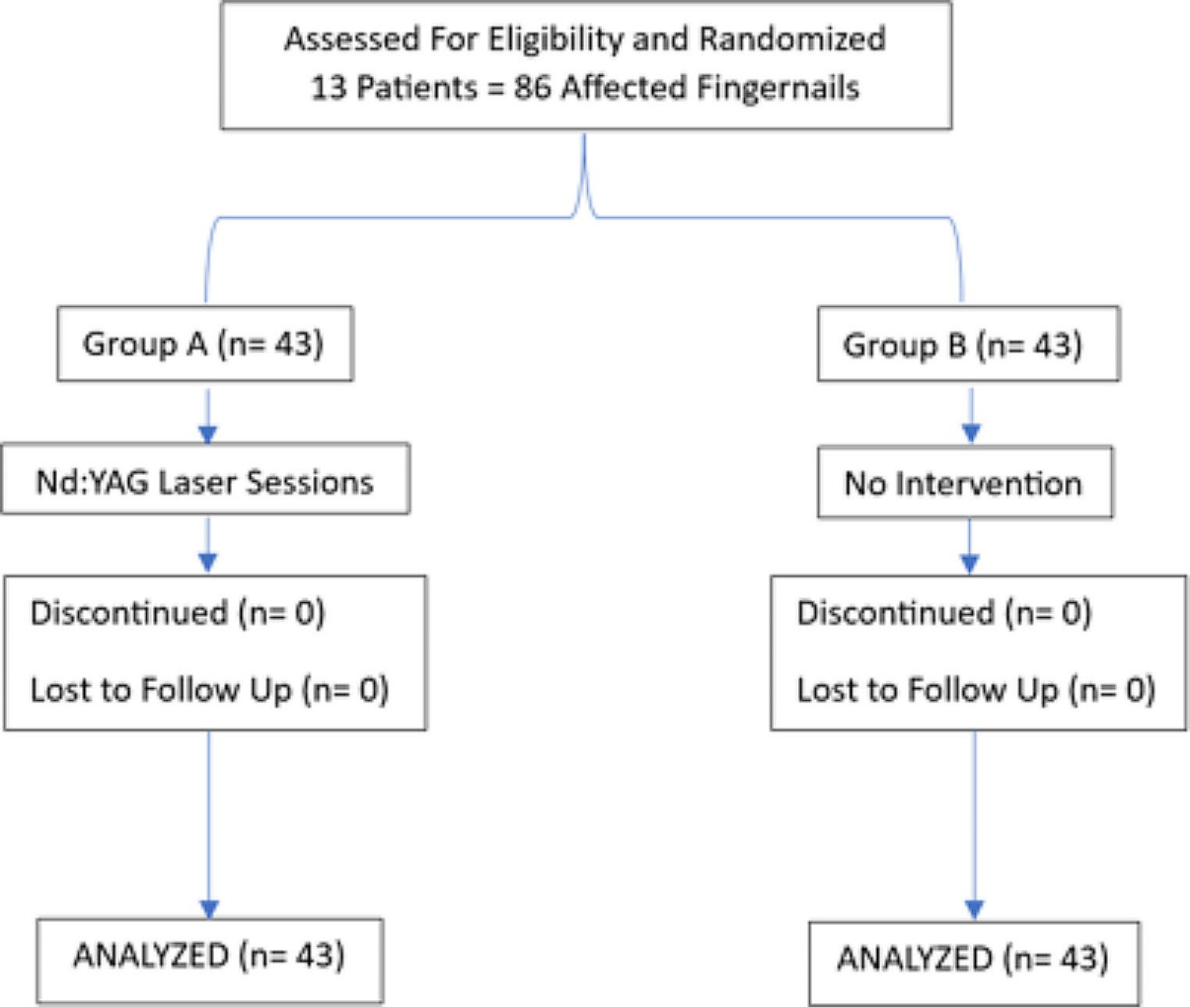
Patients flow chart

For all included recruits, each fingernail was dealt with as a different case to a total of 86 fingernails. The card randomization procedure was used to distribute the affected fingernails to receive either long-pulsed Nd: YAG 1064 nm laser treatment or serve as control. Accordingly, 43 fingernails were included in group A to receive long-pulsed Nd: YAG 1064 nm laser treatment and 43 fingernails were included in group B to serve as control (Fig. 1).

### Long-pulsed 1064 nm nd: YAG Laser

Long-pulsed Nd: YAG 1064 nm laser was applied using the (Cynosure®, INC, 5 Carlisle Road, Westford, MA 01886, USA). Patients received 3 sessions with 1-month intervals, with 2 passes of energy 20 Joules/cm^2^, pulse duration 10ms and spot size 5 mm, together with continuous cold air cooling.

### Assessment methods

#### Clinical assessment

Assessments were done at the baseline, 1 month from last treatment session (EOS) and 2 months later at the last follow up (FU).

For clinical scoring of nail psoriasis, we used the 32-points target NAPSI (tNAPSI) scoring system where nails were examined for pitting, leukonychia, lunular red spots, crumbling, onycholysis, subungual hyperkeratosis, salmon patches and splinter hemorrhages [13]. Nail pits were counted and analyzed separately at every evaluation point.

Assessment included photography using a digital camera (Canon DS126291, CANON INC. Taiwan) and a dermoscope (Dermlite DL4, USA) to be evaluated by two blinded dermatologists experienced in psoriasis management at FU according to the physician’s global assessment of fingernail psoriasis score that ranged from 0 to 4 corresponding to clear, minimal, mild, moderate and severe [14].

#### Ultrasonographic assessment

A musculoskeletal ultrasound evaluation was carried out at the baseline and FU using a GE LOGIQ P6 device (Model 183995SUO, India) in conjunction with a high-frequency broadband linear array transducer (10-13 MHz). The settings for Doppler were a 500 Hz pulse repetition frequency, with color gain optimized for optimal sensitivity while minimizing excessive color noise. The patients were seated with their forearms, hands, and fingers in a neutral and relaxed posture on a table, and their fingernails were scanned longitudinally. To ensure an adequate acoustic interface and prevent compression of the structures, a thick gel layer was applied.

The severity of psoriatic nail changes was assessed using the Wortsman’s classification (0–4), which includes Grade 0 (normal nail plate consisting of two parallel hyperechoic bilaminar bands together with a virtual anechoic space between them), Grade I (focal hyperechoic involvement of the ventral nail plate without involvement of the dorsal nail plate), Grade II (continuous loss of the borders of the ventral nail plate), Grade III (wavy plates without blurring of both plates), and Grade IV (loss of definition of both nail plates) [15].

The thickness of the hypoechoic nail bed was measured as the maximum distance between the ventral plate of the nail and the edge of the phalangeal bone, while the thickness of the isoechoic area of the nail matrix was measured at the proximal end of the nail bed.

The vascularity of the nail bed was assessed using the power Doppler (PD) technique on a scale of 0 to 3, with 0 indicating no PD signal (normal), Grade I indicating a signal present in less than 25% of the examined area, Grade II indicating a signal present on 25% but less than 50% of the examined area, and Grade III indicating a signal present on 50% or more of the examined area [16].

#### Patients’ self-assessment

Patients were asked to report the degree of pain they felt during the first session for Nd: YAG laser through the pain visual assessment score (0–10) where “0” =no pain, “1–3” =mild pain, “4–6” =moderate to severe, “7–9” =very severe and “10” =worst pain possible [17].

### Statistical analysis

Sample size: Using the Power Analysis and Sample Size (PASS) 11th release [18], a sample size of 21 nails in each group was calculated to have at least an 80% power to detect the differences at α = 0.050 between the Nd: YAG laser and control groups with total score mean ± SD at FU of 16.0 ± 5.8 and 22.85 ± 9.0, among Nd: YAG laser and placebo sides respectively [11]. We increased the sample for possible attrition up to 40 nails in each of the two studied groups.

Data were coded and entered using the Statistical Package for the Social Sciences (SPSS) version 28, 2021 (IBM Corp., Armonk, NY, USA). Data was summarized using mean, standard deviation, median, minimum and maximum in quantitative data and using frequency (count) and relative frequency (percentage) for categorical data. For comparison of serial measurements within each patient the non-parametric Wilcoxon signed rank test was used, comparisons between quantitative variables were done using the non-parametric Mann-Whitney test [19]. For comparing categorical data, Chi square (χ2) test was performed. Exact test was used instead when the expected frequency is less than 5 [20]. Testing for inter-rater and intra-rater reliability was done using the Intra Class Coefficient (ICC) and Cronbach’s alpha reliability coefficient with their 95% confidence interval (95%CI) [21]. *P*-values less than 0.05 were considered as statistically significant.

## Results

Thirteen patients (86 fingernails), seven males and six females; completed the study and were analyzed. Patients ages ranged from 18 to 63 years with a median of 31 years, the duration of skin psoriasis ranged from 1 to 35 years with a median of 7 years and the duration of nail psoriasis ranged from 4 months-14 years with a median of 1.5 years. Six patients were cigarette smokers. Three patients had a family history of psoriasis. Urban residents were nine and four were rural residents. Twelve of the thirteen patients (92.3%) were manual workers.

### Changes in target NAPSI and pitting

At baseline, we did not detect any significant differences between the studied groups regarding medians of tNAPSI scores and counted numbers of pits. Comparison between Nd: YAG laser and control groups at the end of FU did not show any statistically significant differences regarding the medians of tNAPSI score or pits count, and this was the case with both blinded dermatologists and ultrasonographic assessments as well.

**Fig. 2:**
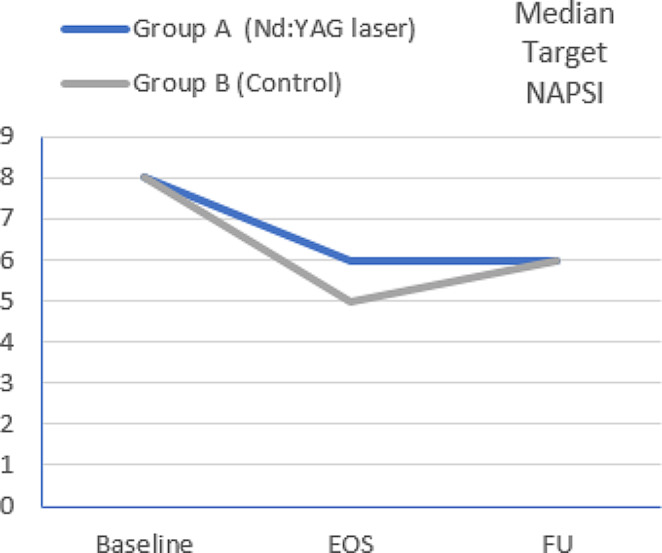
Changes in median tNAPSI scores over time among studied groups

**Fig. 3:**
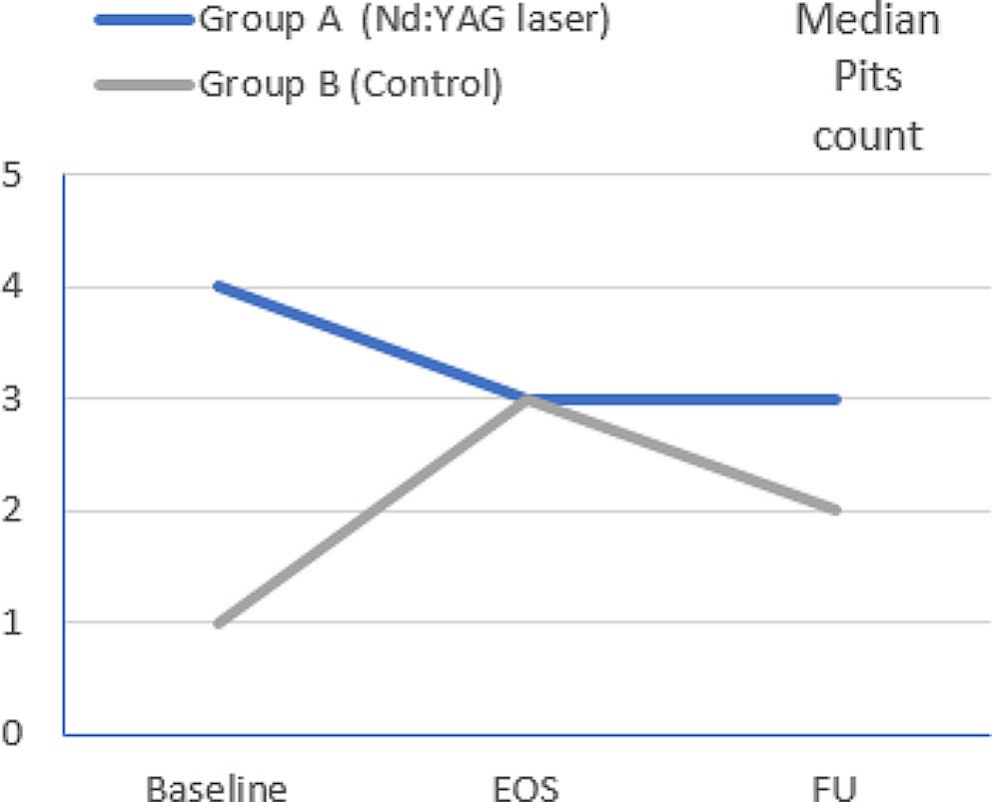
Changes in median pits count over time among studied groups

**Fig. 4:**
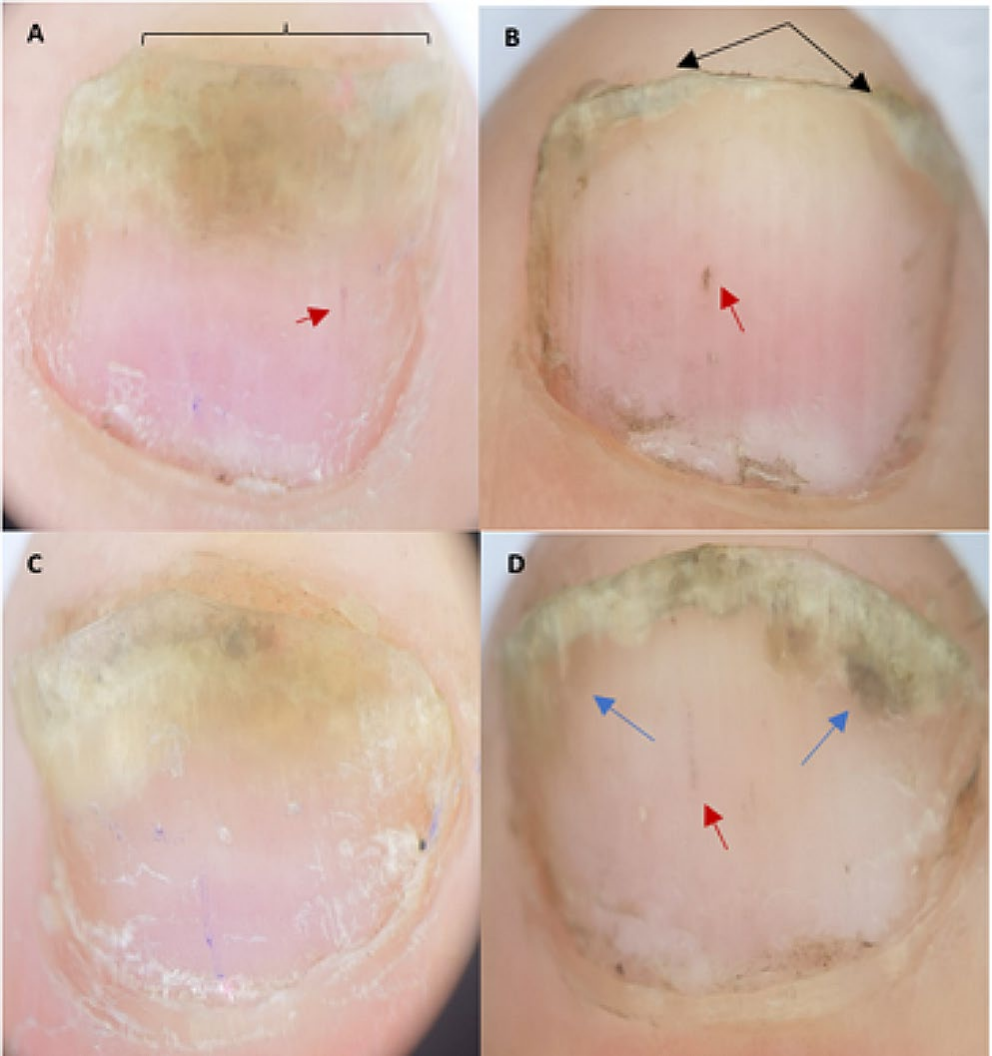
Clinical assessment of laser-treated and control nails of one patient at baseline and follow up

However, when groups were analyzed separately, there was a statistically significant difference in tNAPSI score between baseline and FU in group A (*p =* 0.001), while in group B this was non-significant (*p =* 0.054). The median percentage of improvement of tNAPSI score at FU in group A and B were 33.33% and 12.50%, respectively (Table 1; Figs. 2, 3 and 4).

**Table 1:** Changes in target NAPSI scores and pits count at baseline and FU among studied groups

		Group A (Nd: YAG)		Group B (control)		*p* value
		Median (range)		Median (range)		
**Target NAPSI**	**Baseline**	8 (2–18)	*p* value = 0.001*	8 (0–15)	*p* value = 0.054	0.501
	**FU**	6 (0–14)		6 (0–16)		0.801
	**(%) Improvement**	33.33%		12.50%		0.247
**Pits count**	**Baseline**	4 (0–13)	*p* value = 0.514	1 (0–9)	*p* value = 0.181	0.133
	**FU**	3 (0–18)		2 (0–15)		0.558
	**(%) Improvement**	33.33%		28.57%		0.557

FU: Follow up after 3 month of last laser session. Data is presented as median and range (non-normally distributed). *p* < 0.05* is considered significant

**Fig. 5:**
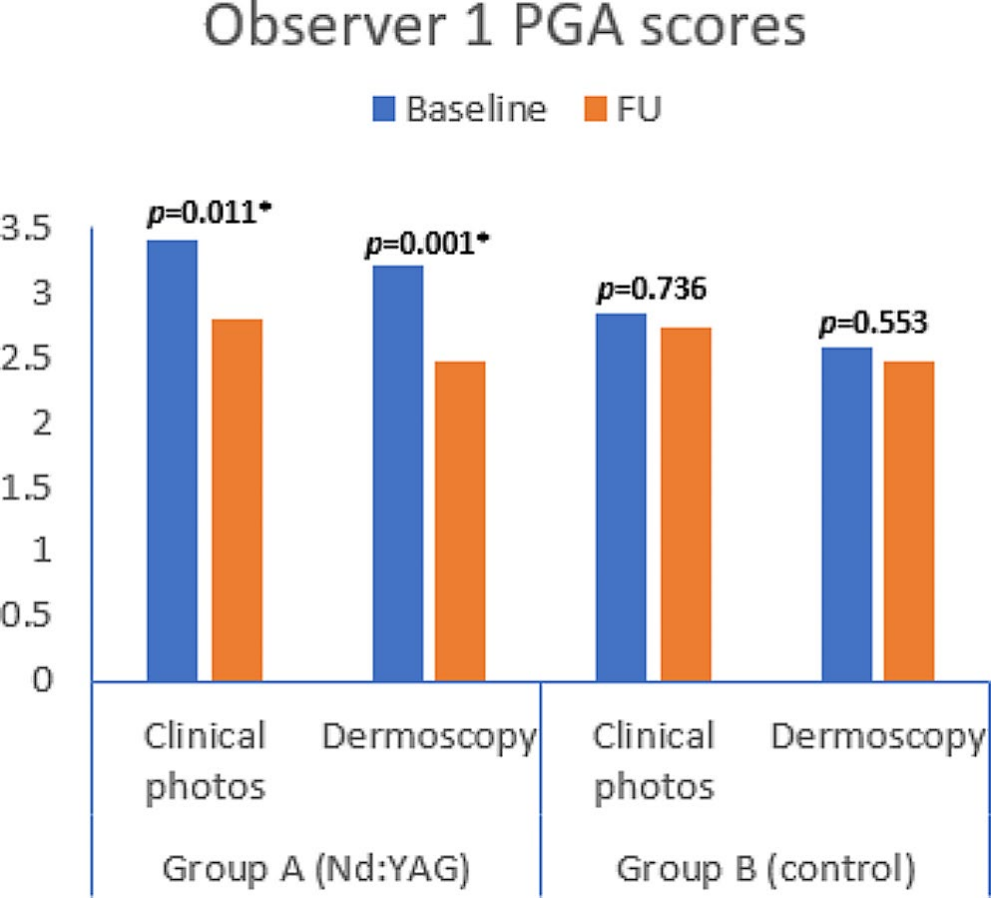
Physicians’ global assessment by first blinded dermatologist at FU

**Fig. 6:**
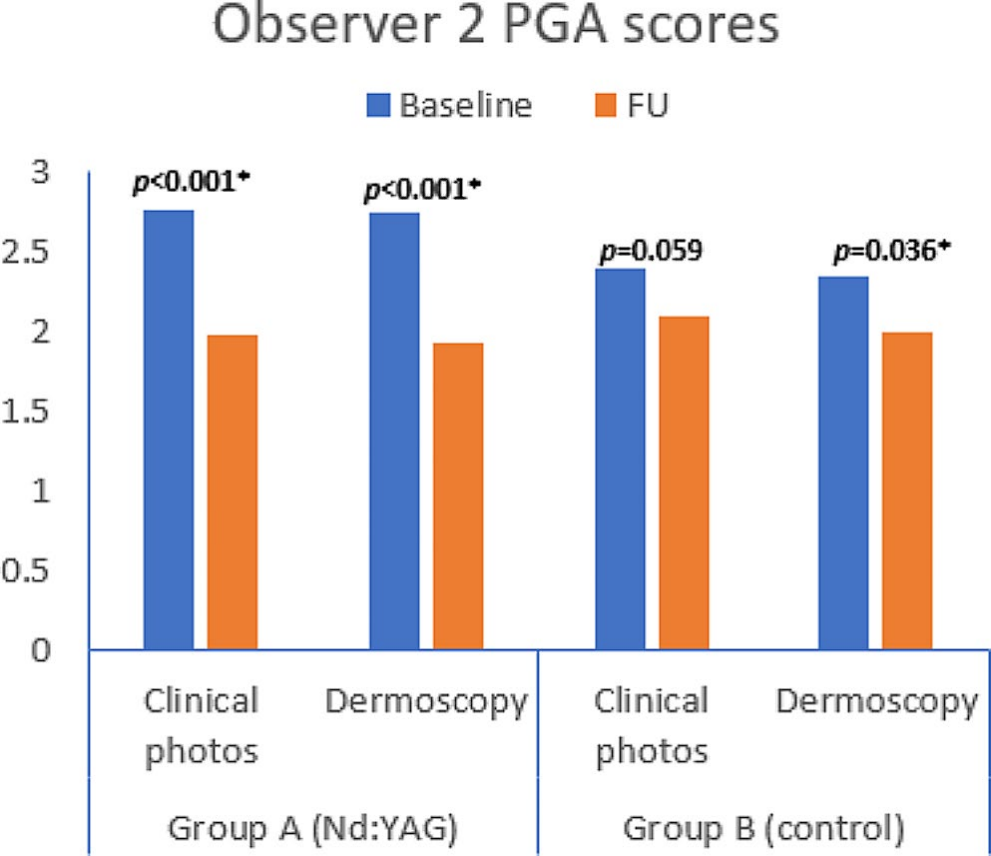
Physicians’ global assessment by second blinded dermatologist at FU

Also, blinded dermatologists’ photographic assessment of clinical as well as dermoscopic photos showed statistically significant improvement at FU in group A when analyzed separately, while this was not the case in the control group except for dermoscopic evaluation of the 2nd observer (Figs. 5 and 6).

### Changes in ultrasonography

**Fig. 7:**
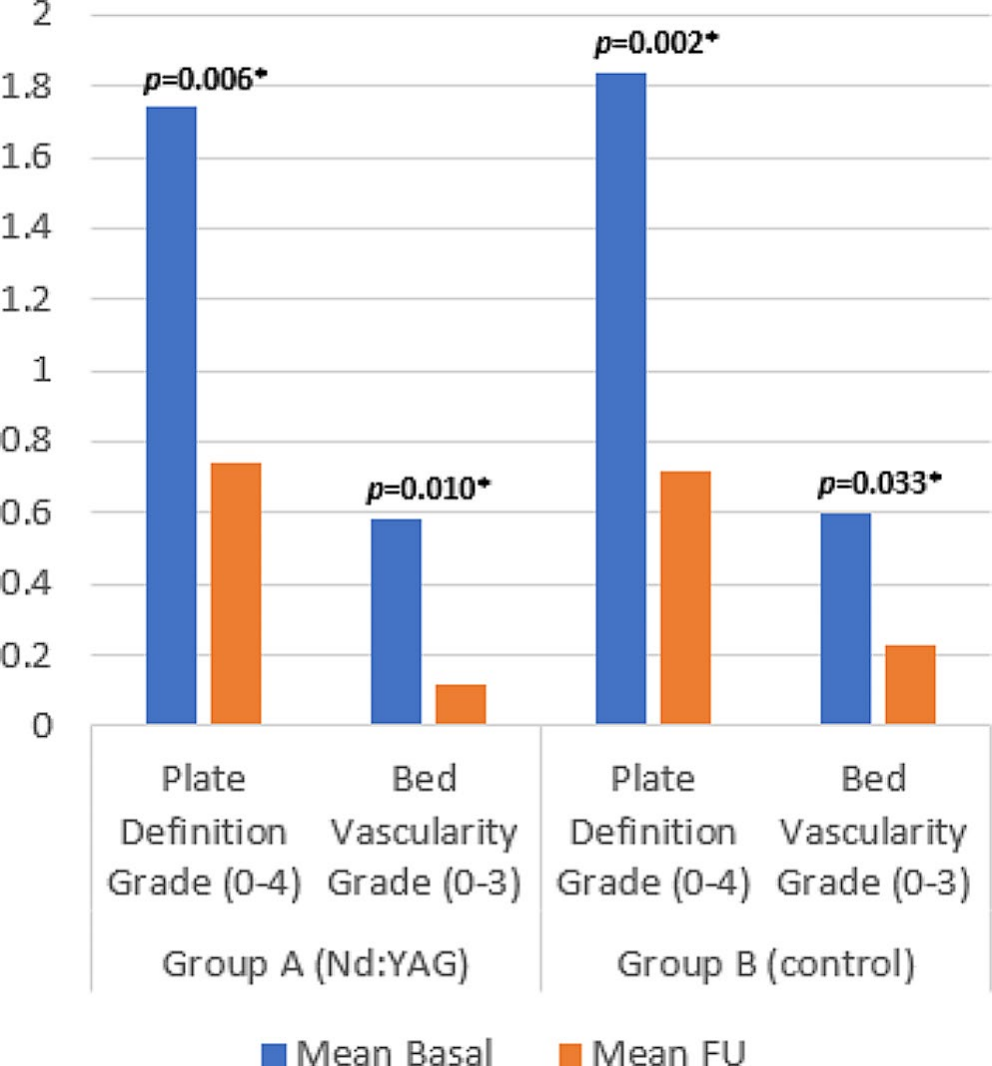
Ultrasonographic assessment at baseline and FU of nail plate definition grades (0–4) and nail bed vascularity grades (0–3)

**Fig. 8:**
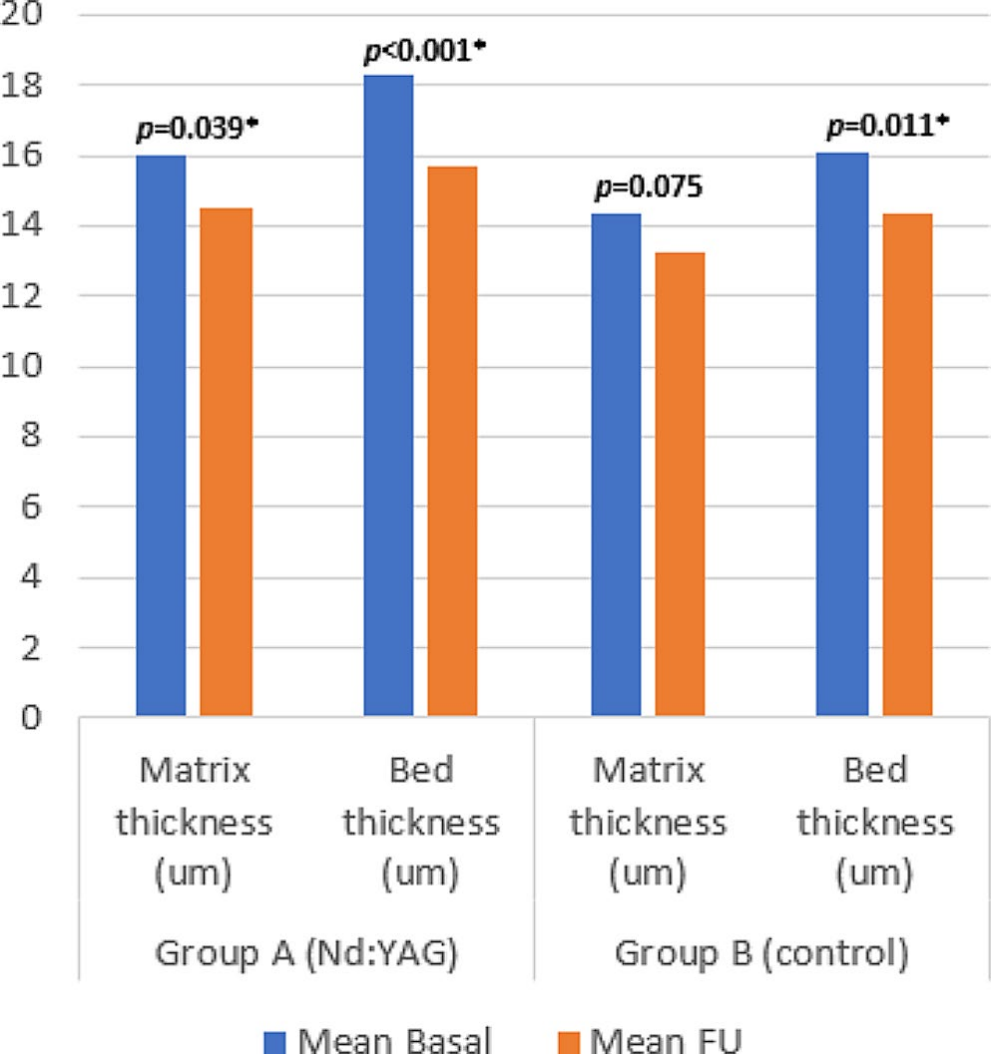
Ultrasonographic assessment at baseline and FU of nail matrix and bed thickness (um)

**Fig. 9:**
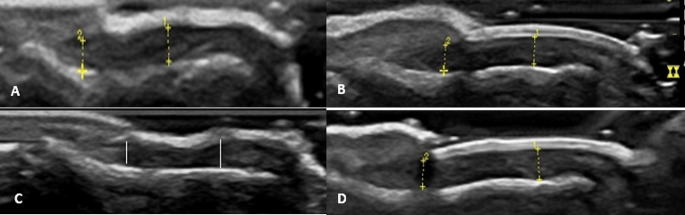
Ultrasonographic assessment of laser-treated and control nails of another patient at baseline and follow up

Ultrasonographic assessment of the Nd: YAG and control groups detected a statistically significant improvement in the median nail plate definition grade (*p* = 0.006 and 0.002, respectively) at end of FU period. Median nail matrix thickness showed significant reduction in the Nd: YAG treated group (*p* = 0.039) but not in the control group. Median nail bed thickness was reduced significantly in both Nd: YAG and control groups (*p* < 0.001, 0.011, respectively) and bed vascularity grade also showed significant improvement in both groups (*p* = 0.010, 0.033, respectively) (Table 2; Figs. 7, 8 and 9).

**Table 2:** Analysis of baseline and follow up values of ultrasonographic findings among studied groups

		Group A (Nd: YAG)		Group B (control)		*p* value
		Median (range)		Median (range)		
**Nail Plate Definition**	**Baseline**	1 (0–4)	*p* = 0.006*	1 (0–4)	*p* = 0.002*	0.883
	**FU**	0 (0–4)		0 (0–4)		0.733
	**(%) Improvement**	100%		100%		0.736
**Nail Matrix Thickness (um)**	**Baseline**	16 (9–25)	*p* = 0.039*	14 (08–22)	*p* = 0.075	0.029*
	**FU**	14 (08–24)		14 (8–23)		0.108
	**(%) Improvement**	12%		6%		0.870
**Nail Bed Thickness (um)**	**Baseline**	18 (10–31)	*p* < 0.001*	16 (8–25)	*p* = 0.011*	0.034*
	**FU**	15 (9–23)		14 (8–23)		0.087
	**(%) Improvement**	18%		16%		0.383
**Nail Bed Vascularity**	**Baseline**	0 (0–3)	*p* = 0.010*	0 (0–3)	*p* = 0.033*	0.966
	**FU**	0 (0–1)		0 (0–3)		0.666
	**(%) Improvement**	100%		100%		0.186

FU: Follow up after 3 month of last laser session. um: Micrometer. Data is presented as median and range (non-normally distributed). *p* < 0.05* is considered significant

The median degree of pain assessed through the pain visual assessment score (from 0 to 10) after the first session with the long-pulsed Nd: YAG laser was 2 (0–4).

## Discussion

Treatment of nail psoriasis include topical, intra-lesional and systemic therapies selected according to the disease severity, its impact on the patient’s quality of life and comorbidities [5]. The penetrability of topical agents into the nail bed and/or matrix is essential to achieve therapeutic effect [6]. Nd: YAG laser, based on selective photo-thermolysis of hemoglobin, targets the dermal vasculature of the nail bed; the site of new changes occurring in psoriatic nail lesions [9–11]. In the current work treatment of NP with Nd: YAG laser was associated with significant improvements in tNAPSI, PGA and US measurements of matrix thickness in comparison to untreated nails with no adverse events reported in any of our patients.

The improvement of NP with Nd: YAG treatments we observed as well as previous investigators [9–11, 22] can be attributed to its ability to penetrate down to 5–6 mm in the tissue thus reaching the nail bed vascularity deep enough to target the abnormally increased vasculature in nail psoriasis by selective photo-thermolysis of hemoglobin [23, 24]. In addition, Nd: YAG laser reduces T cytotoxic cells in the epidermis, and T helper cells and CD3 + lymphocytes in the dermis, which in turn normalizes the epidermal proliferation and improve the clinical signs of psoriasis [10].

It is worth mentioning that Elwan et al. (2021) did not find significant improvements with Nd: YAG laser Vs controls in a left to right sided comparison of NP treatment after 6 month. Unlike the current study, the latter authors included toenail psoriasis, used NAPSI score and applied one pass with spot size 2.5 mm with laser energy of 110 J/cm^2^ to 130 J/cm^2^. They assumed that this non-significance was because they treated both finger-and toenails and that toenails may be more resistant than fingernails.[25, 26]

In the current study, we observed a non-significant spontaneous improvement in our “*untreated*” control group as regards tNAPSI score and this was also observed in the blinded physicians’ PGA score. Surprisingly, this non-significant clinical improvement was associated with significant ultrasonographic changes suggestive of a tendency towards normalization of nail plate definition, nail bed thickness and vascularity. Such observations are not unusual, as the course of NP is unpredictable and it may improve spontaneously or show unexpected remissions and exacerbations.[25–29] These improvements we report for the control group may also be attributed to the small sample size, or the systemic effect of locally targeted laser and thermal energies. Anders et al. (2015) and Khalkhal et al. (2020) described that laser irradiation can alter cellular metabolism and cellular functions in the body. They reported that local photo-biomodulation can produce systemic effects and that local irradiation produces distant effects. In a study performed on rats, the application of laser on a standardized skin wound had systemic effects on the wounds located distally from the point of laser application.[30–32]

### Limitations

The limitations in the present study were the relatively small sample. Also, the fact that 92.3% were manual workers and housewives using unprotected bare hands may have hindered the therapeutic effects desired from treatment.

## Conclusion

In conclusion, Nd: YAG laser can be considered safe, efficacious, local method of treating nail psoriasis. Ultrasonography is a useful noninvasive diagnostic and follow up tool that helps in detection of even subclinical changes. Our results also confirm that, nail psoriasis although a treatment challenge; may sometimes improves spontaneously.

### Recommendation

Further larger scale studies for extended time periods are needed to verify our results and investigate the usefulness of US monitoring of nail changes in the treatment of NP.

## Data Availability

No datasets were generated or analysed during the current study.

## Declarations

### Clinical trials registration

The trial was registered on ClinicalTrials.gov with the reference number “NCT05695833”.
